# A Novel Mitochondrial Genome Resource for the Endemic Fish *Gymnodiptychus integrigymnatus* and Insights into the Phylogenetic Relationships of Schizothoracinae

**DOI:** 10.3390/biology14121760

**Published:** 2025-12-09

**Authors:** Yanping Li, Yawen Luo, Yunyun Lv, Yangjia Ou, Ruilin Zhang, Renyi Zhang

**Affiliations:** 1 Fishes Conservation and Utilization in the Upper Reaches of the Yangtze River Key Laboratory of Sichuan Province, College of Fisheries, Neijiang Normal University, Neijiang 641100, China; liyanping_sci@foxmail.com (Y.L.); luoyawen1011@163.com (Y.L.); lvyunyun_sci@foxmail.com (Y.L.); oyj1223@126.com (Y.O.); zhangruilin_ns@126.com (R.Z.); 2 College of Life Science, Neijiang Normal University, Neijiang 641100, China; 3 School of Life Sciences, Guizhou Normal University, Guiyang 550025, China

**Keywords:** Schizothoracinae, Qinghai–Xizang Plateau, mitochondrial genome, phylogenetic relationship, non-monophyletic

## Abstract

*Gymnodiptychus integrigymnatus* is a small endemic fish species found in southwest China. This study aimed to investigate the phylogenetic relationships and mitogenomic characteristics of *G. integrigymnatus*. The complete mitochondrial genome of one individual of *G. integrigymnatus* was sequenced using the Illumina HiSeq X Ten sequencing platform and comprehensively characterized. The mitochondrial genome was 16,714 bp in length, including 13 protein-coding genes (PCGs), 22 transfer RNA (tRNA) genes, 2 ribosomal RNA (rRNA) genes, and a typical control region. The genome composition showed a positive A + T skew (0.029) and a negative G + C skew (−0.198). All tRNAs were predicted to form typical cloverleaf secondary structures, except for tRNA-Ser (GCT), which lacked the DHU stem. Phylogenetic reconstruction based on 13 PCGs revealed that the subfamily Schizothoracinae was not a monophyletic group. Two major clades were identified within this subfamily: one comprising primitive taxa (*Percocypris*, *Aspiorhynchus*, and *Schizothorax*) and the other consisting of specialized and highly specialized groups. *Gymnodiptychus integrigymnatus* was distinct from both its congeners and the specialized clade and was instead recovered within a highly specialized group of the Schizothoracinae subfamily. This study offers novel perspectives on the taxonomy and phylogenetic relationships within the Schizothoracinae subfamily, with a specific focus on *G. integrigymnatus*, thereby enhancing the understanding of the systematics and phylogeny of the Schizothoracinae subfamily.

## Simple Summary

*Gymnodiptychus integrigymnatus*, a member of the subfamily Schizothoracinae, is found exclusively in the streams of the Gaoligongshan Mountains. This study aimed to elucidate the phylogenetic relationships and mitogenomic characteristics of *G. integrigymnatus.* The results indicated that the mitochondrial genome of *G. integrigymnatus* was similar to those of other fish species in terms of gene order, nucleotide composition, codon usage bias, and tRNA gene structure. Furthermore, the phylogenetic tree constructed from the 13 mitochondrial protein-coding genes revealed the Schizothoracinae subfamily as non-monophyletic. Additionally, *G. integrigymnatus* was resolved outside the clade containing its congeners in the genus *Gymnodiptychus*.

## 1. Introduction

The subfamily Schizothoracinae is a special group within the family Cyprinidae that has adapted to the cold-water environments of the Qinghai–Xizang Plateau and its surrounding areas [1,2]. Its unique features, such as scale degeneration and specialized large scales (pelvic scales) located on both sides of the anus and the pelvic fins, make it an ideal model for studying fish adaptation, evolution, and biogeography on the plateau. Cao et al. classified the subfamily Schizothoracinae into three grades: primitive, specialized, and highly specialized Schizothoracine fishes [3]. This classification is based on characteristics such as modifications in scales, pharyngeal teeth, and barbels. This division is closely associated with the adaptive radiation prompted by the sequential uplift of the Qinghai–Xizang Plateau [4,5,6].

The genus *Gymnodiptychus* was categorized as specialized, along with *Diptychus* and *Ptychobarbus*. A morphology-based cladistic analysis by Chen and Chen supported the monophyly of the specialized schizothoracines and the genus *Gymnodiptychus*, thus placing *G. integrigymnatus* within a monophyletic *Gymnodiptychus* [7]. In contrast, a subsequent molecular phylogeny based on the mitochondrial cytochrome *b* gene by He et al. revealed a different scenario, suggesting that the specialized schizothoracines were not monophyletic and *G. integrigymnatus* was not grouped with its nominal congeners but was closely related to the highly specialized Schizothoracine fishes [8]. These results indicate that the morphological interpretations may be influenced by adaptive convergence, whereas single-gene datasets often lack the signal required to firmly establish deep nodes in the phylogeny.

*Gymnodiptychus integrigymnatus* is a specialized Schizothoracine fish endemic in China, with its distribution limited to the streams of the Gaoligongshan Mountains [9]. It is unique in that it has no scales all over its body, retaining only the anal and lateral axillary scales at the base of the pelvic fins. The dorsal side of *G. integrigymnatus* is yellowish brown with scattered black spots, whereas the abdomen is white [2]. The population of this species experiences a continuous decline owing to the vulnerability of its habitat and the adverse effects of anthropogenic activities. It has been listed as a vulnerable species (VU) in the IUCN Red List [10] and presently recognized as a key protected species within the Gaoligongshan Mountain National Nature Reserve. Current research endeavors predominantly concentrate on morphological classification and distribution assessments, whereas investigations at the molecular level, particularly genomic analyses, are comparatively limited.

The mitochondrial genome (mitogenome) is a powerful molecular marker for phylogenetic studies, particularly valued for its maternal inheritance, relatively rapid evolutionary rate, and conserved gene content across animals [11,12]. Therefore, it has been extensively used as a molecular marker for species identification, population genetics, and phylogenetic reconstruction [13,14]. In fishes, the typical mitogenome is a circular molecule of 15–18 kb, including 13 protein-coding genes (PCGs), 2 ribosomal RNA (rRNA) genes, and 22 transfer RNA (tRNA) genes, alongside a control region [15,16]. Compared with single genes, the complete mitogenome offers more comprehensive and dependable phylogenetic signals, rendering it a valuable resource for resolving phylogenetic controversies [17,18]. Thus, it serves as a valuable tool to re-evaluate the conflicting phylogenetic hypotheses surrounding *G. integrigymnatus*. Previous studies largely focused on sequence variation in PCGs; however, recent advancements in DNA sequencing technologies and assembly strategies have made it possible to characterize the mitogenomes of numerous fish species [19,20,21,22]. A deeper analysis of structural features, such as tRNA secondary structure stability and codon usage bias, can provide valuable insights into evolutionary adaptation [23,24,25,26,27]. Investigating these subtle molecular features, particularly in the extremely high-altitude habitats inhabited by Schizothoracinae fishes may provide key insights into potential adaptive evolutionary mechanisms.

This study aimed to analyze the complete mitochondrial genome of *G. integrigymnatus* to fill the gap in the genetic information of this species and address the following key scientific questions: (1) What are the structural features, codon usage patterns, and tRNA secondary structures of the mitochondrial genome of *G. integrigymnatus*? (2) Using complete mitogenome sequences, what are the phylogenetic relationships of *G. integrigymnatus* with other species within the genus *Gymnodiptychus* and the major groups within the subfamily Schizothoracinae? (3) Does the mitogenome phylogeny support the morphological classification of *G. integrigymnatus*, or does it indicate a case of morphological convergence? The findings may provide a genetic foundation for further investigations into the taxonomy, population genetics, adaptive evolution, and conservation management of *G. integrigymnatus*, as well as the phylogenetic relationships of species within Schizothoracinae.

## 2. Materials and Methods

### 2.1. Specimen Collection, DNA Extraction, and Sequencing

One individual of *G. integrigymnatus* was collected in August 2024 from Tengchong County, Yunnan Province, China (25°25′24″ N, 98°39′8″ E). The specimen was preserved in anhydrous ethanol and deposited in the School of Life Sciences, Guizhou Normal University (GZNUSLS202408797). The experiments involving *G. integrigymnatus* in this study were performed in compliance with the national standards for laboratory animal care and treatment. Total genomic DNA was extracted from the muscle using the DNeasy Blood & Tissue Kit (Qiagen Inc., Hilden, Germany). Its integrity, purity, and concentration were evaluated using an Agilent 5400 fragment analyzer (Agilent Technologies, Santa Clara, CA, USA). Sequencing was performed on total genomic DNA without prior mitochondrial enrichment. The DNA sample was tested and then fragmented using a Covaris focused-ultrasonicator. Subsequently, a DNA library was prepared through terminal repair, A-tail addition, sequencing adaptor addition, purification, and PCR amplification. The concentration of the library was measured with a Qubit 2.0 fluorometer (Life Technologies, Singapore). The inserted fragments of the library were analyzed using an Agilent 2100 bioanalyzer (Agilent Technologies, Santa Clara, CA, USA). Sequencing was conducted on an Illumina HiSeq X Ten sequencing platform at the DNA Stories Bioinformatics Center in Chengdu, Sichuan, China. The mitochondrial genome sequences were subsequently bioinformatically sorted and assembled, as described in the following section.

### 2.2. Sequence Analyses

Quality control and filtering of the raw dataset were performed as follows. Raw sequencing reads in the BCL format were converted into FASTQ files using Bcl2Fastq v.2.20.0. The quality of the raw reads was assessed with FastQC v. 0.11.4 [28]. Adapter sequences, low-quality bases, and undetermined bases were then filtered using SOAPfilter v.2.2 software with the following criteria: (1) removal of reads containing adapter sequences; (2) discarding paired-end reads if >3% of bases in either read were undetermined (N); and (3) removal of paired-end reads if >50% of bases in either read had a quality score below 3. This process yielded high-quality, clean data for subsequent assembly. The mitogenome clean data were assembled using GetOrganelle v. 1.7.7.0 [29] with default parameters. Subsequently, the assembled mitogenome sequence was annotated using the MitoAnnotator tool on the MitoFish homepage [30]. tRNA genes were further identified and their secondary structures predicted using MITOS v 2.1.9 on the Galaxy platform [31]. The secondary structures of tRNAs were drawn using MITOS. The base composition, codon usage, and relative synonymous codon usage (RSCU) for each PCG were calculated using PhyloSuite v1.2.3 [32]. The sequences were further analyzed by determining A + T skew and G + C skew using standard formulas: the A + T skew was defined as (A% − T%)/(A% + T%), whereas the G + C skew was defined as (G% − C%)/(G% + C%) [33].

### 2.3. Phylogenetic Analyses

The mitochondrial genomes from 75 species within the subfamily of Schizothoracinae, along with 8 outgroup taxa, comprising 124 samples, were retrieved from the NCBI database to investigate the phylogenetic relationships between *G. integrigymnatus* and the subfamily Schizothoracinae (Table S1). These sequences were subsequently integrated with the newly assembled complete mitochondrial genome of *G. integrigymnatus* obtained in this study to conduct phylogenetic analyses. The shared 13 concatenated PCGs were extracted and recombined to construct a matrix using PhyloSuite [32]. MAFFT v7.0 [34] was used to align the nucleotide sequences of the concatenated supergene comprising 13 PCGs with default parameters. The optimal partition strategy and the best-fit evolution model of each partition were estimated using PartitionFinder v2.1.1 [35] under the corrected Akaike information criterion and a greedy search scheme. The best-fit scheme defined 10 partitions (P1–P10). The GTR + I + G model was assigned to eight partitions: P1 (ATPase 6), P3 (COXI, COXIII), P4 (COXII), P5 (Cyt *b*), P6 (ND1, ND3, ND4), P7 (ND2), P9 (ND5), and P10 (ND6). The remaining partitions were P2 (ATPase 8; HKY + I + G) and P8 (ND4L; GTR + G). Bayesian inference (BI) analysis was implemented using MrBayes v3.2.6 [36] under the unlinked branch lengths of each partition scheme. Each dataset was analyzed with two independent runs of four Markov chain Monte Carlo chains (one cold chain and three heated chains), which were run for two million generations and samples were saved for every 100 generations. Convergence was confirmed using an average potential scale reduction factor of 1.004 and an average estimated sample size of 266.06, after discarding the first 25% of samples as burn-in. Maximum likelihood (ML) analysis was performed using IQ-tree v.1.6.8 [37] with 10,000 bootstrap replicates using the ultrafast bootstrapping algorithm and a maximum of 1000 iterations. All analytical tools were integrated in PhyloSuite with default parameters. The phylogenetic trees were visualized using FigTree v1.4.2 (http://tree.bio.ed.ac.uk/software/figtree/, accessed on 26 November 2025).

## 3. Results

### 3.1. Mitogenome Organization and Nucleotide Composition

**Figure 1:**
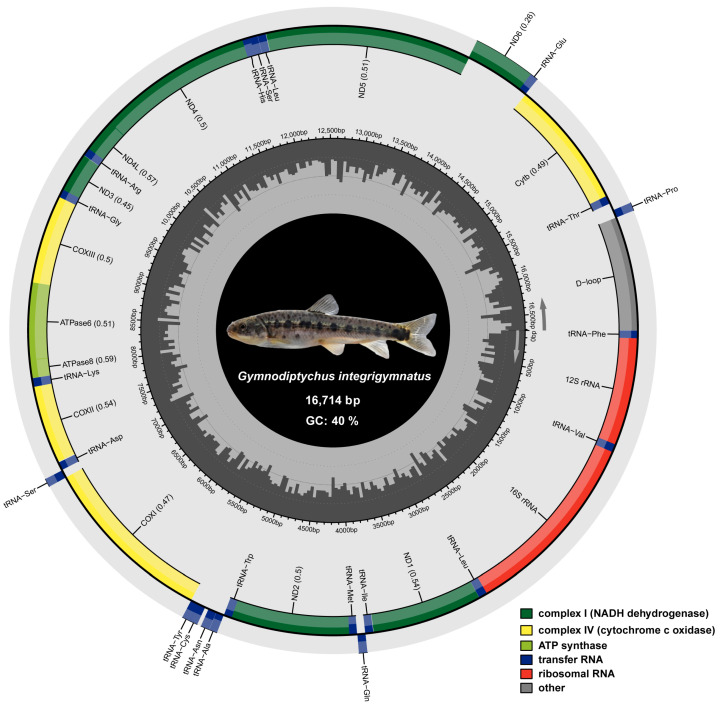
Mitogenome circle map of *G. integrigymnatus*. Genes encoded on the heavy and light strands are depicted inside and outside the circular mitochondrial genome map, respectively. Protein-coding genes associated with NADH dehydrogenase are highlighted in green, whereas those encoding cytochrome c oxidases are highlighted in yellow. Genes for ATP synthase are highlighted in light green. Transfer RNA genes are marked in blue, and ribosomal RNA genes are marked in red. The control region, corresponding to the D-loop, is colored gray.

The total length of *G. integrigymnatus* mitogenome sequence was 16,714 bp. The complete mitogenome of *G. integrigymnatus* was annotated and submitted to GenBank. It comprised 13 typical PCGs, 22 tRNA genes, 2 rRNA genes, and 1 control region (Figure 1 and Table 1). Most PCGs were located on the heavy strand (H-strand), with only the ND6 gene on the light strand (L-strand). Among the 22 tRNA genes, 14 were on the H-strand [tRNA-Phe, tRNA-Val, tRNA-Leu (UUR), tRNA-Ile, tRNA-Met, tRNA-Trp, tRNA-Asp, tRNA-Lys, tRNA-Gly, tRNA-Arg, tRNA-His, tRNA-Ser (AGY), tRNA-Leu (CUN), and tRNA-Thr]. The remaining eight tRNA genes were on the L-strand [tRNA-Gln, tRNA-Ala, tRNA-Asn, tRNA-Cys, tRNA-Tyr, tRNA-Ser (UCN), tRNA-Glu, and tRNA-Pro] (Figure 1 and Table 1).

A total of 127 intergenic nucleotides were distributed across 15 intergenic spacer regions in the mitogenomes of *G. integrigymnatus*, with lengths varying from 1 to 65 bp. The largest spacer was observed between the tRNA-Thr and tRNA-Pro. Moreover, 6 overlapping regions were identified, collectively encompassing 22 bp and ranging in length from 1 to 7 bp. The most extensive overlap was observed between the ATPase 8 and ATPase 6 genes, as well as between the ND4L and ND4 genes. The remaining gene pairs were closely arranged with no intervals or overlaps.

The overall nucleotide composition of the mitogenome sequence was 30.9% A, 29.1% T, 23.9% C, and 16.0% G; it had an A + T-rich feature (60.0%). The A + T contents of PCGs, rRNAs, tRNAs, and the D-loop region all exceeded 50%. The D-loop region, recognized as an A + T-rich segment, exhibited the highest A + T content at 67.5%. Furthermore, the A + T content at the first (50.7%), second (59.8%), and third (72.2%) codon positions of the PCGs exhibited statistically significant differences. The *G. integrigymnatus* mitogenome was slightly A-skewed and moderately C-skewed, with a positive A + T skew of 0.029 and a negative G + C skew of −0.198 (Table 2). The A + T skew of the mitogenome, tRNAs, rRNAs, and three PCGs (ATPase 8, COXII, and ND2) were all positive, whereas the control region, 10 PCGs, and concatenated PCGs were negative. The A + T skew was negative for the second codon positions of PCGs but positive for the first and third codon positions. Regarding G + C skew of the mitogenome, positive values were observed only for tRNAs, first codon positions of PCGs, and ND6. In contrast, negative G + C skew values were found for rRNAs, concatenated PCGs, second and third codon positions of PCGs, 12 individual PCGs, and the control region (Table 2).

### 3.2. Protein-Coding Genes

The mitogenome of *G. integrigymnatus* included 13 PCGs, ranging from 165 to 1821 bp in length (Table 1). The A + T content of its PCGs was 60.9%. All the PCGs started with the typical ATG initiation codons, except for COXI, which started with GTG. In contrast, the stop codon usage varied across genes: ND1, ATPase 8, and ND3 employed the complete stop codon TAG; COXI, ND4L, ND5, and ND6 used TAA as the complete stop codon; ATPase 6 and COXIII had the incomplete stop codon TA; and the remaining genes (ND2, COXII, ND4, and Cyt *b*) terminated with the incomplete stop codon T (Table 1).

**Figure 2:**
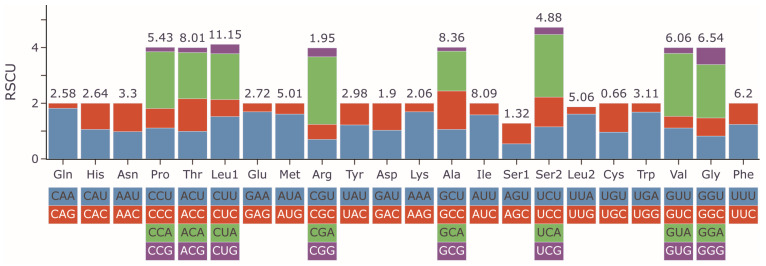
RSCU of the mitogenome for *G. integrigymnatus*. The frequency of each amino acid (top number, %) is presented above the stacked columns.

RSCU values were calculated to measure the codon usage bias (Figure 2 and Table S2). The 13 PCGs encoded 3793 amino acids (except for 7 stop codons). The three most utilized codons in *G. integrigymnatus* mitogenome were AUU, CUA, and UUA. Synonymous codons included 29 preferred codons, each exhibiting an RSCU value greater than 1; the maximum observed value was CGA-Arg (2.43), whereas the minimum was GCG-Ala (0.14). Four codons including GUA, UCA, CCA, and CGA demonstrated RSCU values exceeding 2; all of these codons terminated with A. The most frequently encoded amino acid in proteins was arginine (Arg), followed by valine (Val), serine (Ser), and proline (Pro) (Figure 2 and Table S2).

### 3.3. Transfer RNAs, Ribosomal RNAs, and Control Region

**Figure 3:**
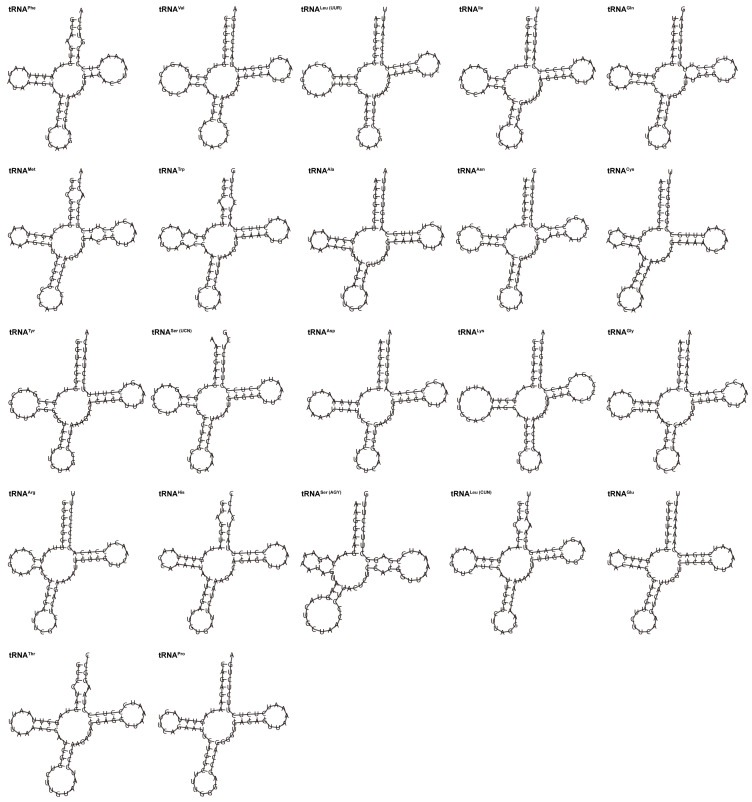
Predicted secondary structures of 22 tRNAs in the mitogenome of *G. integrigymnatus*.

*G. integrigymnatus* mitogenome had 22 tRNA genes, ranging from 66 to 77 bp in length. These tRNAs exhibited an AT bias (57.3%), similar to the overall mitogenomic composition. The A + T and G + C skew values were 0.039 and 0.042, respectively (Table 2). Except for tRNA-Ser (AGY) exhibiting an incomplete dihydrouridine arm (DHU arm), all the tRNAs were folded into typical cloverleaf secondary structures, which comprised four distinct domains (the amino acid arm, the DHU arm, the anticodon arm, and the TΨC stem) (Figure 3). Furthermore, the findings revealed the presence of canonical G-C and A-U base pairs across all tRNA molecules. However, the sequences also had certain instances of non-canonical or mismatched base pairs. For example, 15 of 22 tRNA genes, including tRNA-Phe, tRNA-Val, tRNA-Leu, tRNA-Gln, tRNA-Met, tRNA-Ala, tRNA-Asn, tRNA-Tyr, tRNA-Ser, tRNA-Asp, tRNA-Lys, tRNA-Gly, tRNA-Arg, tRNA-His, and tRNA-Glu, had 29 G-U mismatches in their secondary structures, which formed a weak bond. Five tRNAs, including tRNA-Trp, tRNA-Ser, tRNA-His, tRNA-Leu, and tRNA-Thr, had A-C mismatches in their amino acid acceptor arm. Two A-G mismatches were identified within the amino acid acceptor arm and the dihydrouridine arm of tRNA-Phe and tRNA-Arg, respectively. A single U-C mismatch of tRNA-Met and one U-U mismatch of tRNA-Asn were found in their TΨC stem. Also, a single A-A mismatch was detected within the dihydrouridine arm of the tRNA-Trp molecule.

The mitogenome of *G. integrigymnatus* also contained two rRNAs. 12S rRNA was 953 bp long and positioned between tRNA-Phe and tRNA-Val, whereas 16S rRNA was 1677 bp long and located between tRNA-Val and tRNA-Leu (UUR). The nucleotide composition of the two ribosomal RNAs was determined to be 21.4% T, 23.0% C, 34.1% A, and 21.4% G. The combined A + T content was 55.5%, which was marginally greater than the combined G and C content. The control region in *G. integrigymnatus* was 1010 bp long, with an A + T content of 67.5%; it was situated between tRNA-Pro and tRNA-Phe.

### 3.4. Phylogenetic Relationships

**Figure 4:**
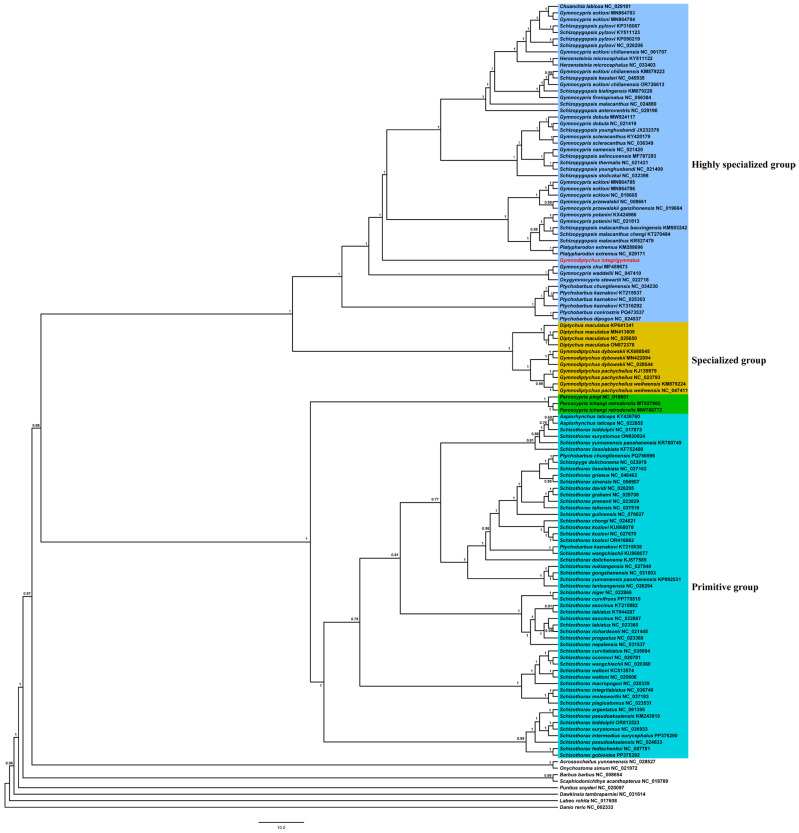
BI phylogenetic tree based on 13 PCGs of 76 Schizothoracinae species and 8 outgroup species. Numbers at nodes represent the posterior probability for BI analysis. The red color words denoted species with novel mitogenomes sequenced in this study.

The phylogenetic tree was reconstructed using the 13 PCGs from 76 species within the subfamily Schizothoracinae, along with eight outgroup species, to determine the phylogenetic position of *G. integrigymnatus* (Table S1). In this study, 2 different methods, ML and BI, were used to construct trees for the 13 concatenated datasets of PCGs, yielding consistent phylogenetic results (Figure 4 and Figure S1). Our analysis of mitogenomes revealed two distinct clades within Schizothoracinae. One clade included *Percocypris*, *Aspiorhynchus*, and *Schizothorax* from the primitive group, whereas the other encompassed all members of the specialized and highly specialized groups. The phylogenetic results indicated that *G. integrigymnatus* did not cluster with other species belonging to the genus *Gymnodiptychus*, but instead grouped with the highly specialized group (Figure 4 and Figure S1). 

## 4. Discussion

### 4.1. Mitochondrial Genome Characteristics of G. integrigymnatus

This study sequenced, annotated, and characterized the complete mitogenome of *G. integrigymnatus*. The results indicated that the mitogenome was a closed double-stranded circular structure, 16,714 bp in length. The length of the mitogenome fell within the range previously reported for the subfamily Schizothoracinae (Table S1). The mitogenome was composed of 37 genes (13 PCGs, 22 tRNAs, and 2 rRNAs) and 1 control region. The genome size, structural organization, and gene composition of the mitogenome of *G. integrigymnatus* were highly conserved compared with those of most previously characterized Cypriniformes and cyprinid species, consistent with the general evolutionary stability of vertebrate mitochondrial genomes [38,39,40,41,42].

The analysis of codon usage for PCGs revealed some notable characteristics. Specifically, GTG served as a start codon for COXI, whereas ATG was the standard start codon for the other genes. Termination codons were of three main types: TAA, TAG, and the incomplete codon T/TA. The occurrence of incomplete termination codons is common in fish mitochondrial genes [13,43,44]. For these incomplete stop codons, the missing nucleotides may be produced through post-transcriptional polyadenylation [45,46]. Phenomena such as AT bias and incomplete stop codons are frequently observed in the mitochondrial genomes of fishes and may be associated with replication and transcription mechanisms, as well as evolutionary selective pressures [47,48].

### 4.2. tRNA Secondary Structure, Codon Usage Preference, and Functional Adaptation

All 21 tRNAs in the mitogenome of *G. integrigymnatus* exhibited a typical cloverleaf secondary structure. However, tRNA-Ser (AGY) was notable for lacking a DHU arm, which is generally present in fish mitogenomes [13,40,43,44]. This phenomenon is regularly observed and may be modified by post-transcriptional editing mechanisms [49]. The secondary structure of tRNAs is fundamentally important for their stability and functional roles. Alterations or deletions of vital elements within the tRNA secondary structure may significantly impact amino acid recognition, consequently disrupting protein synthesis [50]. The tRNA-Ser (AGY) in a majority of vertebrates lacks a DHU arm. Furthermore, the functional consequences resulting from the absence of the DHU arm are mitigated through compensatory interactions [49,51]. Notably, compared with closely related species, specific tRNAs in *G. integrigymnatus*, such as tRNA-Arg and tRNA-Ile, demonstrated a relatively elevated G + C content within their amino acid acceptor arm and TΨC stem. G + C base pairs formed three hydrogen bonds, whereas AT base pairs formed only two hydrogen bonds. This difference means that an increased G + C proportion was generally associated with enhanced thermal stability in these regions, as demonstrated in prokaryotes [52]. This subtle structural modification may constitute a molecular adaptation to low-temperature conditions, considering the cold, high-altitude aquatic environment inhabited by *G. integrigymnatus*. This is consistent with the overarching principle that molecular structures, such as elements of the tRNA apparatus, experience adaptive evolutionary changes to preserve their functionality under thermal stress. This is supported by research on tRNA-modifying enzymes in eukaryotes adapted to cold environments [53,54]. Such an adaptation likely contributes to the preservation of tRNA structural integrity and functionality under cold stress, thereby facilitating the proper progression of mitochondrial translation. This observation offers novel insights into the organelle-level mechanisms underlying environmental adaptation in plateau fish species.

Codon usage analysis across 13 PCGs in *G. integrigymnatus* revealed a strong preference for A- or T-ending codons, such as UUA (Leu), AUU (Ile), and GUA (Val). In contrast, C- or G-ending codons, such as GCG (Ala), GUG (Val), and GGC (Gly), were underrepresented. This A + T bias is consistent with both the overall high A + T content of the mitochondrial genome, suggesting the influence of mutational pressure, and the abundance of tRNAs recognizing these preferred codons, reflecting natural selection for translational efficiency [55]. This pattern, where codon usage co-evolves with the tRNA pool to optimize translation, is a recognized adaptive strategy in mitochondria. Previous studies showed that tRNA genes corresponding to highly used codons were strategically positioned for efficient expression and translational selection dominated codon usage bias in mitochondrial genomes [56,57]. Thus, the observed codon usage pattern likely arose from the interplay of mutation pressure driving A + T enrichment and selection favoring codons matched by abundant tRNAs. Such optimization is particularly crucial in mitochondria, where energy production demands highly efficient translation to minimize metabolic costs and maintain proteostasis [58]. Consistent with this, our analysis of tRNA genes indicated that the tRNA repertoire in *G. integrigymnatus* was adequate to recognize the frequently employed A + T-ending codons. Consequently, the observed codon usage pattern likely resulted from the interplay between mutational pressure, which promoted genome-wide A + T enrichment, and natural selection, which preferentially fixed codons matching highly abundant tRNAs to optimize translation. This may have important adaptive significance in mitochondria, where the energy demand is extremely high. The analysis of RSCU for the PCGs of *G. integrigymnatus* in the present study revealed that the codons GUA, UCA, CCA, and CGA were the most frequently utilized, corresponding to the amino acids Arg, Val, Ser, and Pro, respectively. Previous studies indicated that the codon usage patterns associated with functional gene expression and protein sequence encoding might be influenced by natural selection across species [40,55].

Further, the mitogenome of *G. integrigymnatus* comprises one noncoding region, known as the D-loop, similar to that in other species within the Schizothoracinae subfamily (Table S1). The D-loop has a faster evolutionary rate and is frequently utilized as a marker to assess and compare population genetic diversity, genetic structure, and phylogenetic relationships within Schizothoracinae [59,60].

### 4.3. Reevaluation of the Phylogenetic Relationships of the Subfamily Schizothoracinae

The phylogenetic relationships of the Schizothoracinae subfamily have long been a key area of research in the study of fishes on the Qinghai–Xizang Plateau. Previous studies have focused on the phylogenetic relationship of the Schizothoracinae subfamily using single or multiple genes, and mitochondrial genome sequences [4,61,62]. However, the lack of the unique species, *G. integrigymnatus*, has limited our comprehensive understanding of the phylogenetic relationship of the Schizothoracinae subfamily. The results of phylogenetic studies have indicated that the Schizothoracinae subfamily is not a monophyletic group. This result aligns with previous findings [61,63]. *G. integrigymnatus* does not belong to the genus *Gymnodiptychus*. This implies that the traditional classification of Schizothoracine fishes based on shared morphological traits such as “pelvic scales” may not actually reflect descent from a common recent ancestor. The observed non-monophyly within this group is most plausibly attributed to convergent evolution in morphological traits. Distinct lineages within the Cyprinidae family have independently developed analogous features, such as pelvic scales and exposed body regions, in response to the selective pressures of the fast-flowing, cold aquatic environments characteristic of the Qinghai–Xizang Plateau. These features, previously regarded as synapomorphies defining the Schizothoracine subfamily, are, in fact, homoplastic traits that have arisen multiple times independently. Two hypotheses may explain this pattern: (1) Taxonomic revision hypothesis: The morphological classification of *G. integrigymnatus* within the genus *Gymnodiptychus* may be based solely on certain ancestral traits or characteristics resulting from parallel evolution. Genetically, it exhibits a closer association with the genus *Gymnocypris* or its related taxa. Consequently, forthcoming taxonomic revisions may require a comprehensive reassessment of the systematic placement of *G. integrigymnatus*. (2) Ancient hybridization and introgression hypothesis: The formation of *G. integrigymnatus* might have involved historical hybridization events. Its nuclear genome might have originated primarily from the ancestor of the genus *Gymnodiptychus*, whereas the mitochondrial genome (maternally inherited) was derived from a lineage similar to the ancestor of the genus *Gymnocypris*. This led to a conflict between the mitochondrial genome–based phylogenetic tree and the morphology-based classification system, which was likely more influenced by nuclear genes. In summary, it suggests that the classification of *G. integrigymnatus* requires further in-depth investigation. This study provides a valuable mitochondrial genome resource promoting research on the taxonomy and molecular phylogeny of the Schizothoracinae subfamily.

### 4.4. Importance of Safeguarding and Prospective Developments

This study presents the first comprehensive mitochondrial genome reference sequence for *G. integrigymnatus*. This genomic may be instrumental in future investigations of population genetic diversity and in the application of DNA barcoding techniques aimed at addressing illegal trade [64,65]. Furthermore, elucidating the phylogenetic placement of this species holds immense importance for advancing the understanding of adaptive radiation processes and the mechanisms underlying biodiversity formation within the Schizothoracinae subfamily. Considering the endangered status of *G. integrigymnatus*, the genetic information generated in this study provides a valuable scientific foundation for the conservation and sustainable management of its germplasm resources [66].

Considering the intricate evolutionary history of fishes belonging to the Schizothoracinae subfamily, the dramatic environmental changes on the plateau have imposed similar natural selection pressures on various fish lineages, resulting in widespread morphological convergence. This phenomenon, which has been shown to mislead taxonomy in Schizothoracine fishes, limits the reliability of inferring phylogenetic relationships based solely on morphological traits [67]. Therefore, future investigations should prioritize the integration of nuclear gene data by applying of transcriptome sequencing or reduced representation genome sequencing methodologies to acquire a comprehensive set of nuclear gene markers. This approach can facilitate the construction of species trees and enable the validation of findings derived from maternally inherited mitochondrial genomes in the present study. Moreover, expanding taxonomic sampling by intensifying the collection and genomic sequencing of the Schizothoracinae subfamily and closely related taxa within the Cyprinidae family is essential for accurately resolving divergence times and elucidating the evolutionary trajectories of each lineage. Future studies should focus on elucidating the genetic underpinnings of key morphological traits by examining the genes responsible for key features, such as scale development, and their evolutionary histories. This may allow for the testing of hypotheses regarding convergent evolution from a developmental biology standpoint.

## 5. Conclusions

This study was novel in successfully sequencing and reporting the complete mitogenome of *G. integrigymnatus*. We characterized the structure of the mitogenome and found that it shared common features with other fishes belonging to the Schizothoracinae subfamily, including gene order and nucleotide composition, and focused on the codon usage bias and tRNA structure. We clarified the phylogenetic status of *G. integrigymnatus* by constructing phylogenetic trees using ML and BI methods based on the 13 PCGs. The phylogenetic analysis indicated that the Schizothoracinae subfamily was not a monophyletic group. *G. integrigymnatus* does not belong to the genus *Gymnodiptychus* but belongs to a highly specialized group within the Schizothoracinae subfamily. Future studies should integrate nuclear genomic data to distinguish between homology and convergent similarity. This study provides valuable genetic resources for understanding the phylogeny and biogeography of the Schizothoracinae subfamily, lying a foundation for the conservation genetics of *G. integrigymnatus*.

## Figures and Tables

**Table 1:** Mitogenome organization of *G. integrigymnatus*.

Gene	Strand	Position		Size (bp)	Intergenic Nucleotide	Codon	
		From	To			Start	Stop
tRNA-Phe	H	1	68	68	0		
12S rRNA	H	69	1021	953	–1		
tRNA-Val	H	1021	1092	72	0		
16S rRNA	H	1093	2769	1677	0		
tRNA-Leu (UUR)	H	2770	2845	76	1		
ND1	H	2847	3821	975	3	ATG	TAG
tRNA-Ile	H	3825	3896	72	–2		
tRNA-Gln	L	3895	3965	71	2		
tRNA-Met	H	3968	4036	69	0		
ND2	H	4037	5081	1045	0	ATG	T
tRNA-Trp	H	5082	5152	71	1		
tRNA-Ala	L	5154	5222	69	1		
tRNA-Asn	L	5224	5296	73	33		
tRNA-Cys	L	5330	5395	66	–1		
tRNA-Tyr	L	5395	5465	71	1		
COXI	H	5467	7017	1551	0	GTG	TAA
tRNA-Ser (UCN)	L	7018	7088	71	3		
tRNA-Asp	H	7092	7163	72	6		
COXII	H	7170	7860	691	0	ATG	T
tRNA-Lys	H	7861	7937	77	1		
ATPase 8	H	7939	8103	165	–7	ATG	TAG
ATPase 6	H	8097	8779	683	0	ATG	TA
COXIII	H	8780	9564	785	0	ATG	TA
tRNA-Gly	H	9565	9636	72	0		
ND3	H	9637	9987	351	2	ATG	TAG
tRNA-Arg	H	9990	10,059	70	0		
ND4L	H	10,060	10,356	297	–7	ATG	TAA
ND4	H	10,350	11,730	1381	0	ATG	T
tRNA-His	H	11,731	11,799	69	0		
tRNA-Ser (AGY)	H	11,800	11,868	69	1		
tRNA-Leu (CUN)	H	11,870	11,942	73	3		
ND5	H	11,946	13,766	1821	–4	ATG	TAA
ND6	L	13,763	14,284	522	0	ATG	TAA
tRNA-Glu	L	14,285	14,352	68	4		
Cyt *b*	H	14,357	15,497	1141	0	ATG	T
tRNA-Thr	H	15,498	15,569	72	65		
tRNA-Pro	L	15,635	15,704	70	0		
Control region	H	15,705	16,714	1010	0		

**Table 2:** Nucleotide composition, A + T skew, and G + C skew of *G. integrigymnatus* mitogenome.

Regions	Strand	Size (bp)	T (U)	C	A	G	A + T (%)	G + C (%)	A + T Skewness	G + C Skewness
Full genome	+	16,714	29.1	23.9	30.9	16.0	60.0	39.9	0.029	−0.198
PCGs	All	11,400	31.9	23.8	29.0	15.3	60.9	39.1	−0.048	−0.218
PCGs	+	10,878	31.5	24.4	29.5	14.7	61.0	39.1	−0.032	−0.248
PCGs	–	522	41.4	11.9	19.0	27.8	60.4	39.7	−0.371	0.401
tRNAs	All	1561	27.5	20.4	29.8	22.2	57.3	42.6	0.039	0.042
tRNAs	+	1002	25.7	22.1	31.9	20.3	57.6	42.4	0.107	−0.042
tRNAs	–	559	30.8	17.5	25.9	25.8	56.7	43.3	−0.085	0.190
rRNAs	All	2630	21.4	23.0	34.1	21.4	55.5	44.4	0.229	−0.037
rRNAs	+	2630	21.4	23.0	34.1	21.4	55.5	44.4	0.229	−0.037
1st codon position	All	3800	23.0	23.7	27.7	25.5	50.7	49.2	0.093	0.037
1st codon position	+	3626	22.4	24.4	28.4	24.7	50.8	49.1	0.117	0.007
1st codon position	–	174	35.1	9.2	13.8	42.0	48.9	51.2	−0.435	0.640
2nd codon position	All	3800	41.5	26.6	18.3	13.6	59.8	40.2	−0.388	−0.325
2nd codon position	+	3626	41.3	26.9	18.7	13.1	60.0	40.0	−0.378	−0.345
2nd codon position	–	174	44.8	21.3	10.9	23.0	55.7	44.3	−0.608	0.039
3rd codon position	All	3800	31.2	21.0	41.0	6.8	72.2	27.8	0.136	−0.513
3rd codon position	+	3626	30.6	21.8	41.4	6.2	72.0	28.0	0.151	−0.557
3rd codon position	–	174	44.3	5.2	32.2	18.4	76.5	23.6	−0.158	0.561
ATPase 6	+	683	34.0	23.1	30.6	12.3	64.6	35.4	−0.052	−0.306
ATPase 8	+	165	27.9	24.2	35.8	12.1	63.7	36.3	0.124	−0.333
COXI	+	1551	31.7	23.9	27.0	17.5	58.7	41.4	−0.079	−0.154
COXII	+	691	29.1	24.3	31.3	15.3	60.4	39.6	0.036	−0.226
COXIII	+	785	30.1	25.0	28.3	16.7	58.4	41.7	−0.031	−0.199
Cyt *b*	+	1141	34.4	22.3	28.8	14.5	63.2	36.8	−0.087	−0.210
ND1	+	975	31.2	26.1	28.1	14.7	59.3	40.8	−0.052	−0.280
ND2	+	1045	27.5	27.8	31.6	13.2	59.1	41.0	0.070	−0.355
ND3	+	351	33.9	22.8	27.4	16.0	61.3	38.8	−0.107	−0.176
ND4	+	1381	31.7	23.7	30.5	14.1	62.2	37.8	−0.020	−0.253
ND4L	+	297	33.7	25.9	23.9	16.5	57.6	42.4	−0.170	−0.222
ND5	+	1821	32.0	23.9	31.0	13.1	63.0	37.0	−0.015	−0.294
ND6	–	522	41.4	11.9	19.0	27.8	60.4	39.7	−0.371	0.401
16S rRNA	+	1677	21.5	21.9	36.0	20.7	57.5	42.6	0.252	−0.028
12S rRNA	+	953	21.3	25.1	31.0	22.7	52.3	47.8	0.185	−0.051
Control region	+	1010	35.6	18.9	31.9	13.6	67.5	32.5	−0.055	−0.163

“+” indicates the heavy strand (H), which corresponds to genes encoded by the heavy strand. “–” indicates the light strand (L), which corresponds to genes encoded by the light strand.

## Data Availability

The mitogenome sequence of *G. integrigymnatus* has been submitted to GenBank (http://www.ncbi.nlm.nih.gov) under the accession no. PX503671.

## Supplementary Materials

The following supporting information can be downloaded at: https://www.mdpi.com/article/10.3390/biology14121760/s1. Figure S1. ML phylogenetic tree based on 13 PCGs of 76 Schizothoracinae species and 8 outgroup species. Numbers at nodes represent the bootstrap values for ML analysis. Table S1. List of species used in phylogenetic analyses based on mitogenomes. Table S2. Relative synonymous codon usage (RSCU) of protein-coding genes in the mitogenome of *G. integrigymnatus*.
