# Supplementary material for: A Novel Mitochondrial Genome Resource for the Endemic Fish Gymnodiptychus integrigymnatus and Insights into the Phylogenetic Relationships of Schizothoracinae
Source: Biology (Basel). 2025 Dec 9;14(12):1760. doi: 10.3390/biology14121760 (PMC12730580; doi:10.3390/biology14121760)
Supplement: Supplementary file 1 [file biology-14-01760-s001.zip › Table S2.pdf]

**Table S2.** Relative Synonymous Codon Usage (RSCU) of protein-coding genes in the mitogenome of *G. integrigymnatus*.

| Codon | Count | RSCU | Codon | Count | RSCU | Codon | Count | RSCU | Codon | Count | RSCU |
|---|---|---|---|---|---|---|---|---|---|---|---|
| UUU(F) | 146 | 1.24 | UCU(S) | 45 | 1.15 | UAU(Y) | 69 | 1.22 | UGU(C) | 12 | 0.96 |
| UUC(F) | 89 | 0.76 | UCC(S) | 42 | 1.07 | UAC(Y) | 44 | 0.78 | UGC(C) | 13 | 1.04 |
| UUA(L) | 165 | 1.61 | UCA(S) | 88 | 2.25 | UAA(*) | 4 | 2.29 | UGA(W) | 99 | 1.68 |
| UUG(L) | 27 | 0.26 | UCG(S) | 10 | 0.26 | UAG(*) | 3 | 1.71 | UGG(W) | 19 | 0.32 |
| CUU(L) | 156 | 1.52 | CCU(P) | 57 | 1.11 | CAU(H) | 53 | 1.06 | CGU(R) | 13 | 0.7 |
| CUC(L) | 63 | 0.61 | CCC(P) | 36 | 0.7 | CAC(H) | 47 | 0.94 | CGC(R) | 10 | 0.54 |
| CUA(L) | 169 | 1.65 | CCA(P) | 105 | 2.04 | CAA(Q) | 89 | 1.82 | CGA(R) | 45 | 2.43 |
| CUG(L) | 35 | 0.34 | CCG(P) | 8 | 0.16 | CAG(Q) | 9 | 0.18 | CGG(R) | 6 | 0.32 |
| AUU(I) | 242 | 1.58 | ACU(T) | 75 | 0.99 | AAU(N) | 61 | 0.98 | AGU(S) | 21 | 0.54 |
| AUC(I) | 65 | 0.42 | ACC(T) | 89 | 1.17 | AAC(N) | 64 | 1.02 | AGC(S) | 29 | 0.74 |
| AUA(M) | 153 | 1.61 | ACA(T) | 126 | 1.66 | AAA(K) | 66 | 1.69 | AGA(*) | 0 | 0 |
| AUG(M) | 37 | 0.39 | ACG(T) | 14 | 0.18 | AAG(K) | 12 | 0.31 | AGG(*) | 0 | 0 |
| GUU(V) | 64 | 1.11 | GCU(A) | 84 | 1.06 | GAU(D) | 37 | 1.03 | GGU(G) | 51 | 0.82 |
| GUC(V) | 24 | 0.42 | GCC(A) | 109 | 1.38 | GAC(D) | 35 | 0.97 | GGC(G) | 40 | 0.65 |
| GUA(V) | 130 | 2.26 | GCA(A) | 113 | 1.43 | GAA(E) | 87 | 1.69 | GGA(G) | 119 | 1.92 |
| GUG(V) | 12 | 0.21 | GCG(A) | 11 | 0.14 | GAG(E) | 16 | 0.31 | GGG(G) | 38 | 0.61 |
