# Supplementary figures and images for: A Novel Mitochondrial Genome Resource for the Endemic Fish Gymnodiptychus integrigymnatus and Insights into the Phylogenetic Relationships of Schizothoracinae
Source: Biology (Basel). 2025 Dec 9;14(12):1760. doi: 10.3390/biology14121760 (PMC12730580; doi:10.3390/biology14121760)

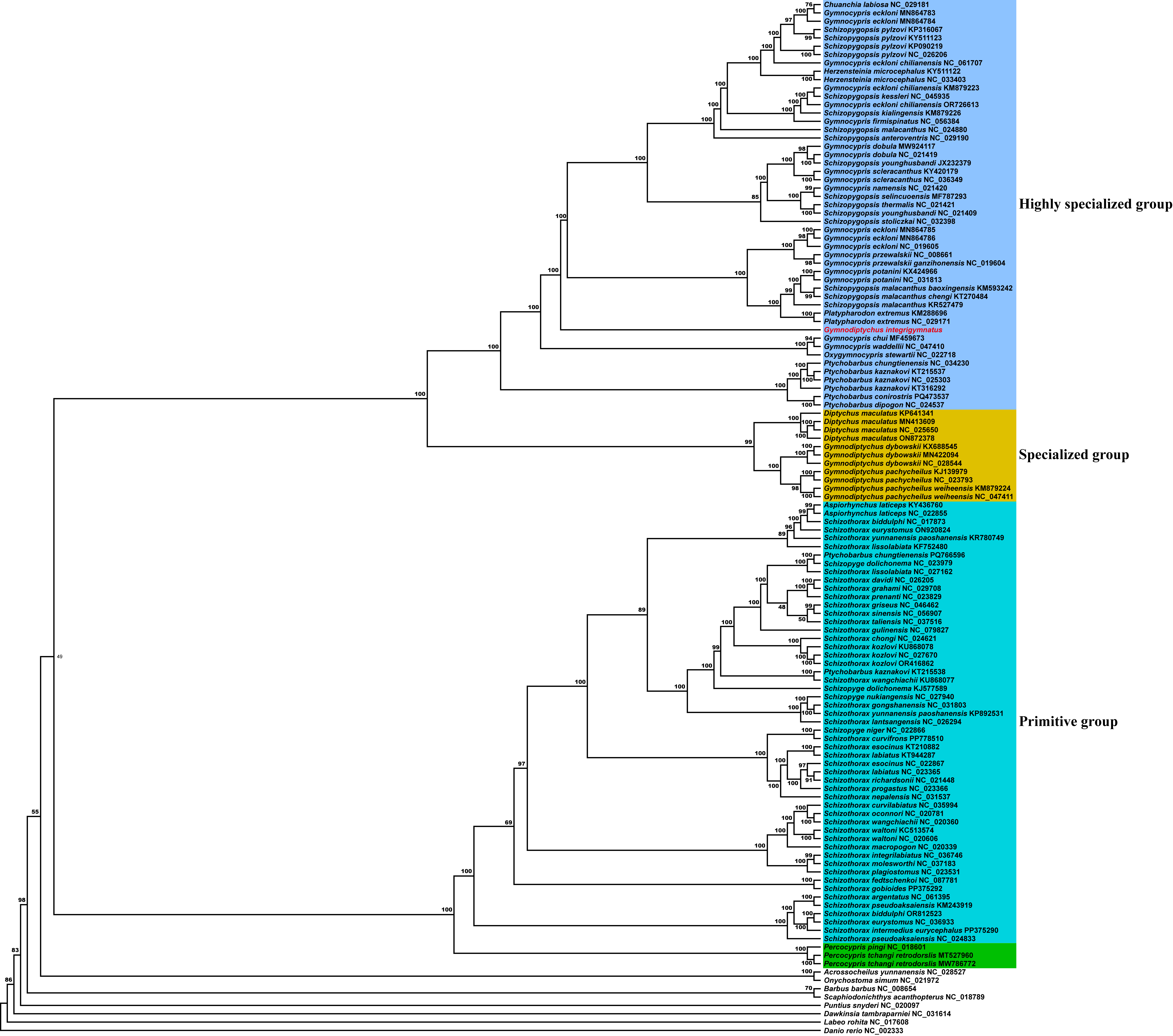

Supplement: Supplementary file 1 [file biology-14-01760-s001.zip › Figure S1.tif]
